# Anti-HER2/neu Antibody Reduces Chemotherapy-Induced Ovarian Toxicity—From Bench to Bedside

**DOI:** 10.3390/biomedicines8120577

**Published:** 2020-12-07

**Authors:** Mattan Levi, Tal Goshen-Lago, Rinat Yerushalmi, Tal Granot, Salomon M. Stemmer, Ruth Shalgi, Irit Ben-Aharon

**Affiliations:** 1 Department of Cell and Developmental Biology, Sackler Faculty of Medicine, Tel Aviv University, Tel Aviv 6997801, Israel; mattanlevi@tauex.tau.ac.il (M.L.); shalgir@tauex.tau.ac.il (R.S.); 2 Division of Oncology, Rambam Health Care Campus, Haifa 3109601, Israel; goshent@gmail.com; 3 Institute of Oncology, Davidoff Center, Beilinson Campus, Rabin Medical Center, Petah-Tiqva 49100, Israel; rinaty@clalit.org.il (R.Y.); tgranot@clalit.org.il (T.G.); stemmer@post.tau.ac.il (S.M.S.); 4 Sackler Faculty of Medicine, Tel-Aviv University, Tel Aviv 6997801, Israel; 5 Rappaport Faculty of Medicine, Technion, Haifa 3200003, Israel

**Keywords:** ovarian toxicity, chemotherapy, anti-HER2/neu, trastuzumab

## Abstract

Background: Trastuzumab, a humanized anti-human epidermal growth factor receptor 2 (HER2/neu) antibody, is considered a standard treatment in addition to chemotherapy in the adjuvant setting for HER2/neu-positive breast cancer, yet its impact on fertility and ovarian reserve remains obscure. We aimed to study the effect of anti-HER2/neu on chemotherapy-induced ovarian toxicity in both clinical and preclinical settings. Methods: We prospectively enrolled breast cancer patients below the age of 42 years who were treated with chemotherapy with or without trastuzumab into the study. Anti-Müllerian hormone (AMH) was measured 6 and 12 months post-chemotherapy as an ovarian reserve indicator. In the animal model, pubertal mice were injected with cyclophosphamide or paclitaxel with or without anti-HER2/neu, or saline, and sacrificed 1 week or 3 months later. Ovarian apoptosis, proliferation and vascularity were measured by immunohistochemistry and ovarian reserve was measured by morphometric analysis and serum-AMH. Results: Thirty-three patients with early breast cancer were enrolled into the study. Nineteen patients had HER2/neu negative cancer and were treated with chemotherapy and 14 had HER2/neu positive cancer and were treated with chemotherapy and trastuzumab. In all patients, AMH levels declined to undetectable values immediately post-treatment, but regained for 57.1% of the HER2/neu positive cohort and 36.8% of the negative cohort (*p* < 0.05). In the preclinical setting, anti-HER2/neu antibody, in combination with chemotherapy, displayed lessened ovarian and vascular damage. Conclusions: Our results indicate that trastuzumab may alleviate chemotherapy-induced ovarian toxicity that may be mediated via its effect on ovarian vasculature.

## 1. Introduction

Breast cancer is the most common cancer among women and one of the leading causes of morbidity and mortality for women worldwide [1]. Cyclophosphamide, which represents the backbone of adjuvant chemotherapy treatment for breast cancer, is considered gonadotoxic and markedly accelerates the rate of age-related ovarian follicle loss, which leads to amenorrhea. As mortality from breast cancer in women under the age of 40 continues to decrease, fertility preservation has become a major survivorship issue [2]. Human epidermal growth factor receptor 2 (HER2/neu) belongs to the receptor tyrosine kinases of the epidermal growth factor family (EGFR), which include four closely related transmembrane tyrosine kinases: ErbB1 (HER1 or EGF receptor), ErbB2 (HER2 or neu), ErbB3 (HER3), and ErbB4 (HER4). HER2 positive breast cancer subtype is relatively more frequent in young women and is associated with aggressive disease and reduced patient survival [3]. The use of trastuzumab, a humanized monoclonal antibody that binds the extracellular, juxtamembranal domain of HER2/neu, in combination with multiple chemotherapeutic agents showed additive and synergistic effects, possibly by increasing susceptibility to chemotherapy-induced apoptosis and slowing down tumor growth and damage repair [4]. We have previously reported that chemotherapy-induced ovarian toxicity may derive from acute vascular insult, demonstrated by decreased ovarian blood flow and diminished post-treatment anti-Müllerian hormone (AMH) levels in a cohort of young breast cancer patients treated with chemotherapy. We observed a differential pattern of ovarian recovery in both ovarian blood flow and biomarkers for gonadal reserve upon the patient’s age, whereas younger patients had regained their ovarian vascular function more than patients that were older than 35 years old [5,6]. Moreover, in a mouse model, we had noted a decrease in ovarian small blood vessels one week after doxorubicin administration manifested by decreased CD34 staining [7] and a sharp elevation in vascular endothelial growth factor (VEGF) level after chemotherapy administration in mice, resulting in later increased ovarian neovascularization. Previous studies have implied that the HER2/neu receptor is expressed on endothelial cells, whereas stimulation of endothelial cells in vitro or in vivo by EGFR resulted in angiogenic responses that were independent of VEGF [3]. Although the synergistic anti- neoplastic role of anti-HER2/neu with chemotherapeutic agents has been extensively studied, its further potential impact on chemotherapy-induced ovarian toxicity is yet to be elucidated. We hypothesized that anti-HER2/neu may modulate the effect chemotherapy exerts on the ovaries and aimed to examine our hypothesis in a translational study—in a prospective observational study and in the preclinical setting in a mouse model.

## 2. Materials and Methods

### 2.1. Experimental Design in Mice

Mature Institute of Cancer Research (ICR) female mice (3 months old; Envigo, Jerusalem, Israel) were housed in air-conditioned, light-controlled animal facilities of the Sackler Faculty of Medicine at Tel-Aviv University. Animal care and all experiments were in accordance with institutional guidelines and were approved by the Institutional Animal Care and Use Committee, Sackler Faculty of Medicine, Tel-Aviv University, ID TAU 01-16-088 approval date 16.08.2016. Females were weighted, injected intraperitoneally twice at one week intervals with saline (control; injected 0.1 mL per 35 g animal); anti-HER2/neu mouse antibody clone 7.16.4 (40 mg/kg; injected 0.1 mL per 35 g animal from a 14 mg/mL stock; catalog number BE0277; BioXCell, West Lebanon, NH, USA); cyclophosphamide (100 mg/kg; Endoxan; injected 0.1 mL per 35 g animal from a 35 mg/mL stock; catalog number D-33790; Baxter Oncology GmbH, Halle, Germany); paclitaxel (8 mg/kg; injected 0.1 mL per 35 g animal from a 3 mg/mL stock; catalog number 33069-62-4; Teva Pharmaceutical Industries, Petah Tikva, Israel); anti-HER2/neu and cyclophosphamide or anti-HER2/neu and paclitaxel. The methodology and doses of anti-HER2/neu mouse antibody, cyclophosphamide and paclitaxel were chosen according to Park et al. [4]. We have previously shown that intraperitoneal administration of chemotherapy in mice is not inferior to intravenous administration by inducing normal tissue toxicity (i.e., gonadal toxicity [6,8,9,10]). We choose to repeat Park et al.’s [4] methodology in mice in order to acquire the additive and synergistic effects of trastuzumab antibody in combination with the chemotherapeutic agents: cyclophosphamide and paclitaxel. Paclitaxel dose was re-calculated and reduced according to our preliminary empirical data in order to meet both gonadotoxic and sub-lethal criteria. Anti-neu antibody (7.16.4) recognizes the juxtamembranal region of rat neu and competes with 4D5, the precursor of trastuzumab, for binding and inhibition of tumor growth. We examined the short- (1 week) and long-term (3 months after drug injection) effects of both chemotherapies. At each time point (1 week and 3 months post injections) mice were sacrificed with isoflurane (Piarmal Healthcare, Mumbai, India) and ovaries were excised, weighed, and further processed.

### 2.2. Patients

Study participants were premenopausal women aged <42 years with regular spontaneous menstruation who were diagnosed with breast cancer and who were chemotherapy-naïve. At the time of enrollment, information regarding demographics, tumor characteristics and treatment protocol was collected. The protocol was approved by the institutional review board (RMC 09-4573; approval date 16.05.2011), and all patients signed an informed consent form. Patients were prospectively enrolled, and serum was collected at baseline, 6 months post-chemotherapy and 12 months post-chemotherapy. Ovarian tissue, obtained from healthy patients < 43 years who underwent partial oophorectomy due to benign conditions and no former exposure to chemotherapy, was retrieved from pathology and processed for histological analyses. Ovarian sections of both cortex and medulla of control and treated patients were processed with hematoxylin and eosin. Sections were stained with anti-CD34 antibody (Abcam, Cambridge, UK, http://www.abcam.com) following standard preparation of deparaffinization and dehydration.

### 2.3. Enzyme-Linked Immunosorbent Assay (ELISA) for AMH

Serum samples for both clinical and preclinical studies were centrifuged (6000 rpm, 10 min, 4 °C) and sera were stored at −80 °C. Measurements of AMH by the designated ELISA kit were according to the manufacturer’s instructions (8; catalog number B13127; Beckman Coulter, Chaska, MN, USA).

### 2.4. Immunohistochemistry (IHC), Terminal Transferase-Mediated Deoxyuridine 5-Triphosphate Nick-End Labeling (TUNEL) and Morphometric Analysis of Ovaries

Sections of paraffin-embedded ovaries were processed as previously described for TUNEL (DeadEnd Fluorometric TUNEL System; catalog number G3250; Promega, Madison, WI, USA) and IHC [9] using primary antibodies: rabbit anti-Ki-67 (1:300 from stock of 0.029 mg/mL; catalog number M3064 Spring Bioscience, CA, USA), rabbit anti-proliferating cell nuclear antigen (PCNA; 1:100 from stock of 0.2 mg/mL; catalog number sc-7907Santa Cruz Biotechnology, Santa Cruz, CA, USA), rat anti-cluster of differentiation (CD34; 1:100 from stock of 1 mg/mL; catalog number CL8927AP; Cedarlane, ON, Canada) and rabbit anti-HER2/neu (1:100 from stock of 1 mg/mL; catalog number E27-63R; SignalChem, Richmond, BC, Canada). We used 1 µg/mL Hoechst 33280; (Sigma Chemicals, St. Louis, MI, USA) for DNA staining, mixed with the following secondary antibodies: HRP-conjugated donkey anti-rabbit (1:200 from stock of 2 mg/mL; catalog number ab6802; Abcam, Cambridge, MA, USA), Alexa-488-conjugated donkey anti-rabbit (1:200 from stock of 2 mg/mL; catalog number ab150065; Abcam), Alexa-555-conjugated donkey anti-rat (1:200 from stock of 2 mg/mL; catalog number ab150153; Abcam). In short, randomly-selected images of 20 transverse sections of ovaries of three mice from each experimental group and from each staining were used for analysis. The average number of TUNEL positive cells or CD34 blood vessels was analyzed by Fiji software (National Institutes of Health, Bethesda, MD, USA). The average numbers of primordial, primary, secondary, and antral follicles were counted as previously described [11].

### 2.5. Quantitative Real-Time PCR (qPCR)

Mice ovarian RNAs were assessed by qPCR as previously described [12]; first-strand cDNA was created by Reverse transcriptase (catalog number 4368814; Applied Biosystems, Foster City, CA, USA), mRNA amount was assessed by SYBR green reagent (catalog number 4309155; SYBR Green PCR Master Mix, Applied Biosystems, Carlsbad, CA, USA). The primers used were as follows: mouse spermatogenesis- and oogenesis-specific basic helix-loop-helix transcription factor 2 (SOHLH2) forward 5′ TCTCAGCCACATCACAGAGG 3′; mouse SOHLH2 reverse 5′ GGGGACGCGAGTCTTATACA 3′; mouse newborn ovary homeobox gene (NOBOX) forward 5′ CATGAAGGGGACCTGAAGAA 3′; mouse NOBOX reverse 5′ GGAAATCTCATGGCGTTTGT 3′; mouse factor in the germline alpha (FIGLA) forward 5′ ACAGAGCAGGAAGCCCAGTA 3′; mouse FIGLA reverse 5′ TGGGTAGCATTTCCCAAGAG 3′. The house-keeping gene selected for the qPCR calibration was hypoxanthine-guanine phosphoribosyl transferase (HPRT1) and the primers used were as follows: HPRT1 forward 5′ CTCATGGACTGATTATGGACAGGAC 3′; mouse HPRT1 reverse 5′ GCAGGTCAGCAAAGAACTTATAGCC 3′.

### 2.6. Statistical Analysis

In this work, all quantitative measurements are presented as mean ± standard error (SEM) after data were evaluated by independent, two-sample *t*-test for unequal sample sizes and unequal variances with a significance of *p* < 0.05 (correlated one-way ANOVA statistical analysis showed similar results).

## 3. Results

### 3.1. Anti-HER2/neu Protect from Ovarian Damage Caused by Chemotherapy in Mice

Mature female mice were injected twice at one week intervals with saline, anti-HER2/neu, cyclophosphamide, paclitaxel while two groups were treated with combination of anti-neu and cyclophosphamide (CTX) or anti-neu and paclitaxel (PTX). Mice were sacrificed after one week for assessment of the gonadal short-term effect, or three months later for assessment of the long-term effect. Our results show that in both general indicators of ovarian reserve and function, the addition of anti-HER2/neu significantly decreased long-term ovarian toxicity caused by chemotherapy.

**Figure 1:**
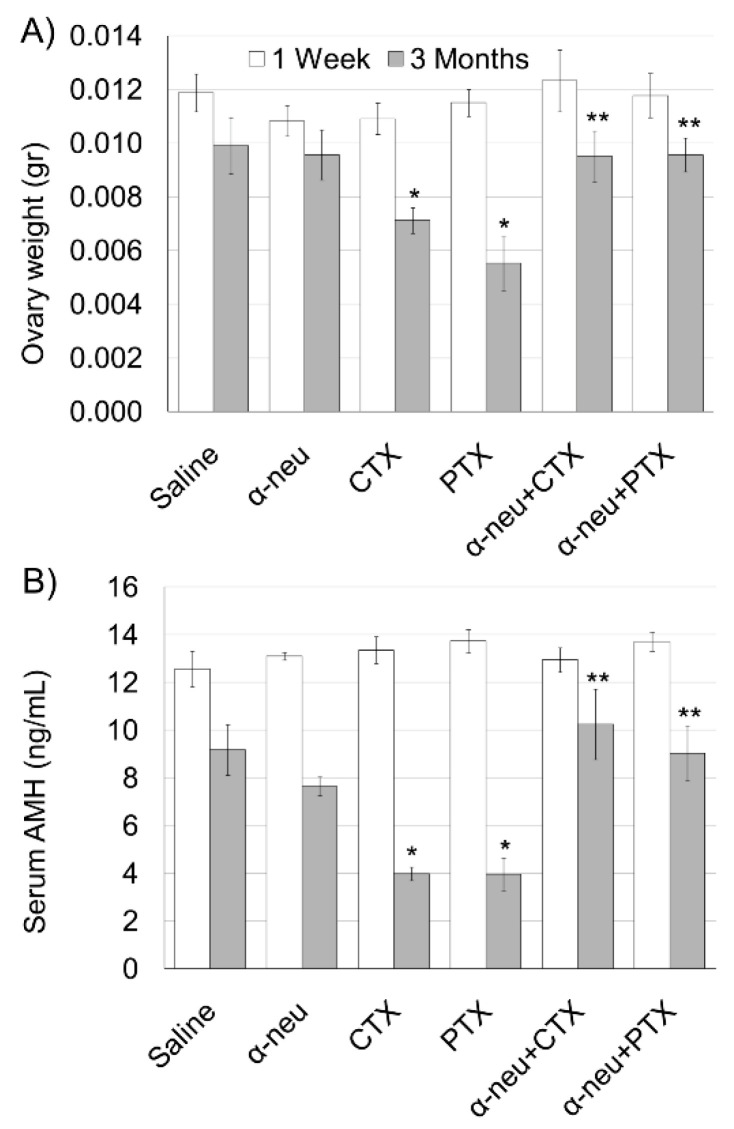
Anti-human epidermal growth factor receptor 2 (HER2/neu) protection from chemotherapy-induced ovarian toxicity in mice. Mature female mice (3 months old; 5 in each group) were injected Intraperitoneal (IP) twice at one week intervals with saline (control), anti-HER2/neu (α-neu; mouse antibody clone 7.140.1; 40 mg/kg), cyclophosphamide (CTX; 100 mg/kg), paclitaxel (PTX; 8 mg/kg), α-neu and CTX or α-neu and PTX and were sacrificed one week (white bars) or three months (grey bars) later. (**A**) Ovary weight and (**B**) serum anti-Müllerian hormone (AMH) were measured. Bars are mean ± SEM. (*)—significantly different from control value (*p* < 0.05). (**)—significantly different from chemo only group (CTX or PTX without anti-neu; *p* < 0.05).

Ovarian weight and serum AMH significantly decreased three months after cyclophosphamide or paclitaxel administration (Figure 1). The combined treatment with anti-HER2 resulted in similar ovarian weight and serum AMH as the control group (Figure 1). Interestingly, anti-HER2/neu alone did not show any effect on both indicators of ovarian reserve and function.

**Figure 2:**
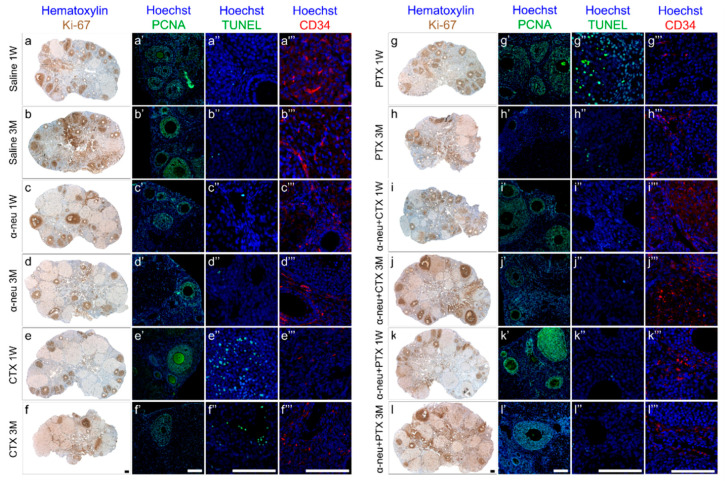
Ovarian proliferation, apoptosis, and vascular state in mice after chemotherapy treatment with or without anti-HER2/neu. Mature female mice were treated as described in the legend of Figure 1. Ovaries were excised from mice one week (1 W) or three months (3 M) after treatment, fixed, paraffin-embedded and serially sectioned for immunohistochemistry, immunofluorescence and terminal transferase-mediated deoxyuridine 5-triphosphate nick-end labeling (TUNEL). Representative bright field images of ovaries stained with Ki-67 (brown; (**a**–**l**)) and representative fluorescence images of ovaries stained against PCNA (green; (**a’**–**l’**)), TUNEL (green; (**a’’**–**l’’**)) or CD34 (red; (**a’’’**–**l’’’**)). Bars = 100 µm.

**Figure 3:**
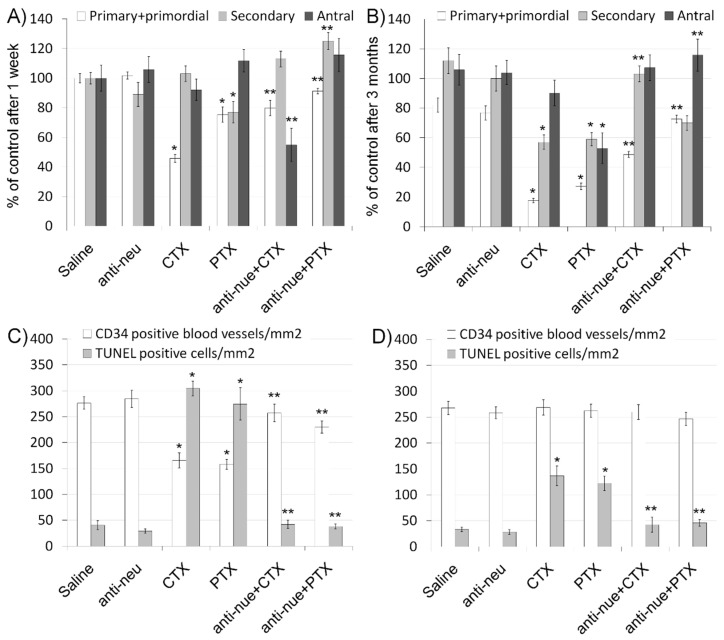
Ovarian follicles in mice after chemotherapy treatment with or without anti-HER2/neu (α-neu). Ovaries were processed as described in the legend of Figure 2 and the average number of primordial and primary (white bars), secondary (grey bars) and antral (black bars) follicles per transverse sections of ovary 1 week (**A**) or three months (**B**) after treatment was measured. Bars are mean ± SEM. (*)—significantly different from control value (*p* < 0.05). (**)—significantly different from chemo only group (CTX or PTX without anti-neu; *p* < 0.05). The average number of CD34 positive blood vessels/mm^2^ (white bars) and TUNEL positive cells/mm^2^ (grey bars) ovary 1 week (**C**) or three months (**D**) after treatment was counted. Bars are mean ± SEM. (*)—significantly different from control value (*p* < 0.05). (**)—significantly different from chemo only group (CTX or PTX without anti-neu; *p* < 0.05).

We further evaluated ovarian function by immunohistochemistry and morphometric analysis. Our results indicate that the ovarian pool of primordial and primary follicles decreased after treatment with cyclophosphamide or paclitaxel after one week (Figure 2 and Figure 3A) as well as after three months (Figure 2 and Figure 3B). This negative effect on ovarian follicular reserve was lessened when anti-HER2/neu was co-injected with these chemotherapeutic drugs. The combination of anti-HER2/neu and chemotherapy had no significant effect on the population of secondary and antral follicles that were also reduced by chemotherapy. Nevertheless, the combined treatment yielded a higher rate of Ki-67 or PCNA positive stained follicles compared with chemotherapy-only groups. Using Ki-67 or PCNA as markers, we could demonstrate increase in proliferation, mainly in secondary and antral follicles (Figure 2).

In order to examine whether the observed protective effect of anti-HER2/neu from chemotherapy-induced ovarian damage involves apoptosis and vascularization, we conducted a TUNEL assay and immunofluorescence of CD34, respectively. As expected, our data demonstrate that administration of cyclophosphamide or paclitaxel resulted in increased apoptosis both at one week (Figure 2 and Figure 3C) as well as at three months (Figure 2 and Figure 3D), whereas co-injection with anti-HER2/neu ameliorated the chemotherapeutic drug effect. Moreover, cyclophosphamide and paclitaxel caused a short-term decrease in CD34-positive blood vessels one week after drug administration (Figure 2 and Figure 3C). The amount of CD34-positive ovarian blood vessels was recovered at three months (Figure 2 and Figure 3D). More importantly, anti-HER2/neu significantly reduced the temporal vascular damage observed at one week of both cyclophosphamide and paclitaxel and displayed a similar amount of CD34-ovarian blood vessels as control (Figure 3C).

**Figure 4:**
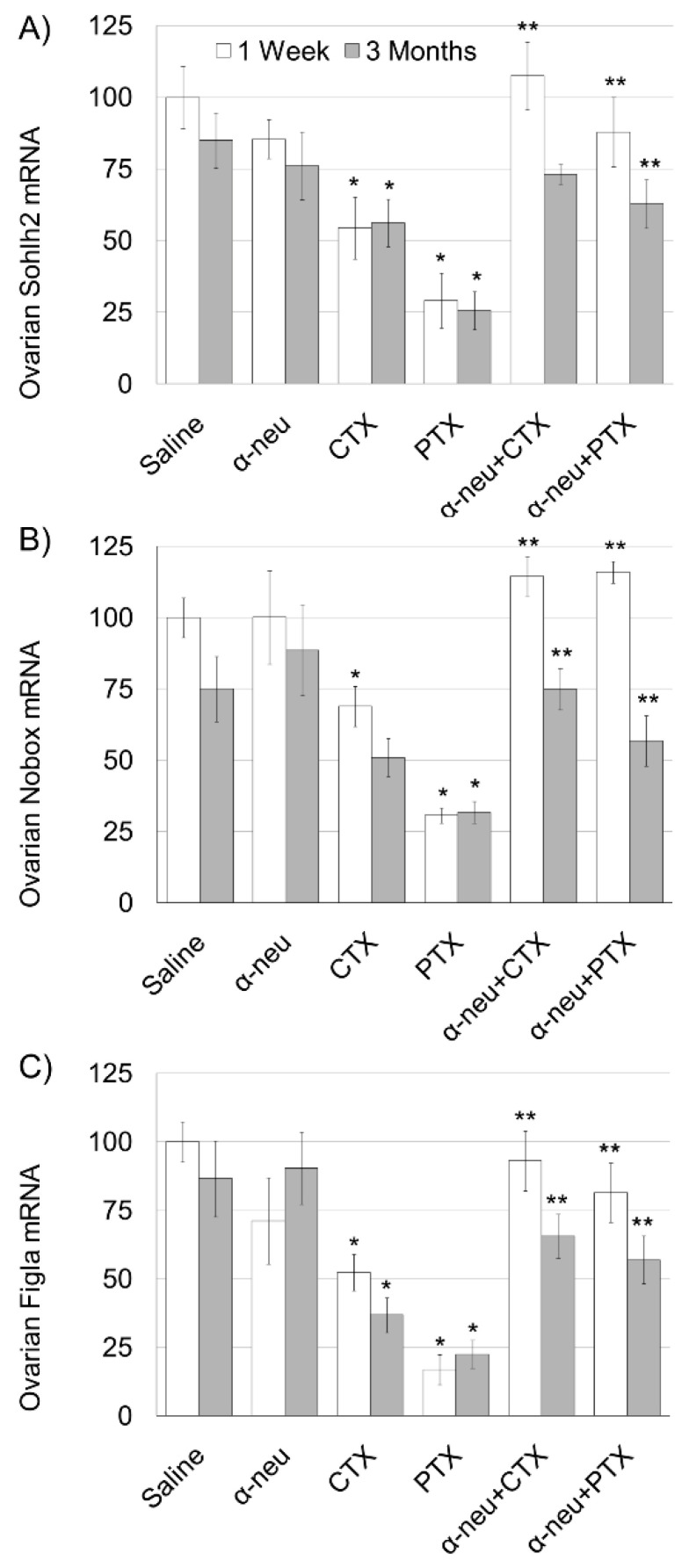
Ovarian reserve in mice after chemotherapy treatment with or without anti-HER2/neu. Mature female mice were treated as described in the legend of Figure 1. Ovaries were excised from mice one week (1 W) or three months (3 M) after treatment and ovarian spermatogenesis- and oogenesis-specific basic helix-loop-helix transcription factor 2 (SOHLH2), newborn ovary homeobox gene (NOBOX) and FIGLA mRNA ((**A**–**C**), respectively) were measured. Bars are % of control ± SEM. (*)—significantly different from control value (*p* < 0.05). (**)—significantly different from chemo only group (CTX or PTX without anti-neu; *p* < 0.05).

Finally, we examined the effect of anti-HER2/neu on ovarian reserve as indicator of the irreversible ovarian damage caused by chemotherapy using quantitative real-time PCR of mRNA of key genes expressed specifically in oocytes of primordial follicles: SOHLH2, NOBOX and FIGLA. Our results indicate a significant decrease in all three indicators one week and three months after cyclophosphamide and paclitaxel administration, with the exception of NOBOX at three months after cyclophosphamide administration (Figure 4). Anti-HER2/neu significantly protected ovarian reserve displaying a significantly higher amount of three indicators both one week and three months after the drug administration, with the exception of SOHLH2, three months after cyclophosphamide administration.

### 3.2. Anti-HER2/neu Lessens Chemotherapy-Induced Ovarian Toxicity in Breast Cancer Patients

Young patients (<42 years) diagnosed with breast cancer were enrolled into the study. Patients presenting with HER2/neu negative cancer were treated with chemotherapy (cyclophosphamide +/− anthracyclines and taxanes; 19 women; median age 34 (25–42)) and patients presenting with HER2/neu positive cancer were treated with chemotherapy and trastuzumab (14 women; median age 36 (26–42)). Patient demographics are presented in Table 1.

**Figure 5:**
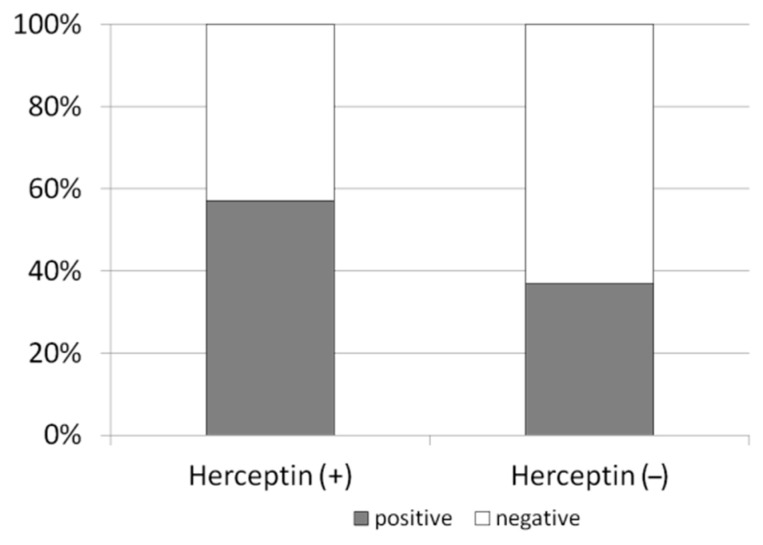
Anti-HER2/neu protection from chemotherapy-induced ovarian toxicity in cancer patients. Serum AMH in breast cancer patients who were treated or not treated with trastuzumab. Positive AMH levels were detected in 57.1% of the Herceptin treated patients compared with 36.8% in the non trastuzumab group (*p* < 0.05).

In all patients, AMH levels declined to undetectable values post-treatment after 6 months. In the HER2/neu positive cohort, AMH level at 12 months post-treatment regained in 8/14 patients (57.1%) and was detectable (Figure 5). In the HER2/neu negative cohort, the AMH at a later time point regained at a significantly lower rate in 7/19 patients (36.8%) compared with the HER2/neu positive cohort (*p* < 0.05). There was no significant difference between the patients who were also treated with Gonadotropin-releasing hormone (GnRH) analogue throughout the treatment period (one in the trastuzumab cohort and two in the chemotherapy cohort).

### 3.3. HER2/neu Expression on Ovarian Vasculature

**Figure 6:**
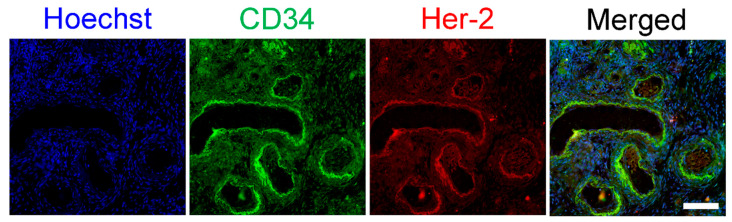
HER2/neu vascular expression in human ovary. Ovaries were fixed, paraffin-embedded and serially sectioned for immunofluorescence. Representative fluorescence images of ovary co-stained against CD34 (green) and Her-2 (Red). Bar = 100 µm.

To determine whether HER2/neu is physiologically expressed in the human ovary and to reveal its possible localization, we used immunofluorescence on ovarian tissue obtained from healthy patients who underwent partial oophorectomy due to benign conditions. Our results show that HER2/neu is expressed in the ovary and is localized at the blood vessels (Figure 6).

## 4. Discussion

Limited information is available concerning ovarian toxicity of targeted agents beyond the damage already caused by chemotherapy [13]. Ovarian function may serve as partial indicator of the damage induced by anti-cancer treatment that could lead to infertility and early menopause. Amenorrhea usually indicates the early chemotherapy toxic effects on the ovaries, but it is also correlated with improved outcomes in patients with hormone receptor-positive breast cancer. Lambertini et al. [13] previously suggested the possible gonadal safety of trastuzumab in the treatment of female cancer patients. In our former study, we have characterized the acute and late effects that several classes of chemotherapies exert on the ovaries in a cohort of young breast cancer patients and revealed that ovarian toxicity was mediated by impairment of ovarian blood flow, which was reversible in some of the patients in an age-dependent manner. There is paucity of data regarding the impact of trastuzumab on fertility. Former evidence from the APT trial, a single-arm phase two adjuvant study of 12 weeks of paclitaxel and trastuzumab followed by nine months of trastuzumab monotherapy, indicates that amenorrhea rates among premenopausal women treated with adjuvant paclitaxel and trastuzumab for early stage breast cancer appeared lower than those seen historically with standard alkylator-based breast cancer regimens [14]. In this study, we aimed to determine the effect of trastuzumab on ovarian reserve in a translational study—in a prospective observational study and in the preclinical setting in a mouse model.

Our results imply that HER2/neu is expressed on ovarian blood vessels and may decrease chemotherapy-induced ovarian toxicity via a possible modulation of the vascular effect exerted by chemotherapy. Our results in a small amount of female cancer patients indicate that AMH, a consensus indicator of ovarian reserve, was higher in the HER2/neu positive cohort compared to the negative cohort. Moreover, anti-HER2/neu significantly reduced the temporal vascular damage of both cyclophosphamide and paclitaxel and exhibited less apoptosis and higher reserve after chemotherapy insult in mice ovaries. Reduced ovarian toxicity observed by combined treatment of trastuzumab and chemotherapy was manifested also in the clinical setting while AMH, a consensus indicator of ovarian reserve, regained to detectable levels post-treatment in a small cohort of breast cancer patients treated with trastuzumab and chemotherapy compared to a small cohort treated with chemotherapy alone. To note, in our former clinical study, we prospectively followed ovarian blood flow using Doppler ultrasound before, during and post-chemotherapy [5,6], while a small cohort had been treated with trastuzumab in addition to chemotherapy. Despite the small sample size (*n* = 7), these patients exhibit a trend in reduced vascular toxicity, as a milder decrease in ovarian blood flow was observed following treatment compared to patients who were treated with chemotherapy only (*p* = 0.068). This observation is in accordance with our current study, which implies that trastuzumab may mediate its possible protective effect via potential modulation of chemotherapy-induced vascular toxicity.

Previous studies have shown that HER2/neu may play a role in regulation of tumor angiogenesis. Trastuzumab may affect VEGF expression and the vascularity of tumors of human Ewing sarcoma cells in vitro and decreased tumor vessel density in vivo [15]. Trastuzumab induces normalization and regression of the vasculature in an experimental human breast tumor that over expresses HER2 in mice and modulates the effects of different pro- and anti-angiogenic factors. Trastuzumab treatment significantly reduces the diameter, volume, and permeability of tumor blood vessels. HER2 signaling is known to control the expression of VEGF and plasminogen activator inhibitor-1 (PAI-1) in vitro. Trastuzumab treatment may affect PAI-1 from the host by modulation of tumor-derived TGF-α. Immunohistochemistry has shown that treatment with trastuzumab induces dynamic changes in the tumor vasculature; in the acute state (14 days), trastuzumab treatment demonstrated CD31-positive blood vessels increase, whereas in the long-term (after 28 days) CD31-positive blood vessels decrease HER2-amplified xenografts [16]. The impact of trastuzumab on tumor microvasculature is still unknown due to imaging limitations, and changes and differences in HER2-amplified breast tumor microvasculature. Decreased VEGF levels in trastuzumab-treated tumors did not change CD31 vessel density [17], indicating that many mechanisms contribute to resistance to trastuzumab, including support of tumor vasculature by tumor cells and upregulation of pro-angiogenic factor heregulin in order to compensate for decreased VEGF production caused by the presence of trastuzumab [18]. Nevertheless, while former literature elaborates on the role of HER2/neu in tumoral angiogenesis, there is a lack of data regarding the role of HER2/neu in normal organ vasculature. Our results suggest that anti-HER2/neu does not increase ovarian vascularity by itself but may protect the chemotherapy-induced obliteration of small ovarian vessels (as demonstrated by reduced CD34 staining).

## 5. Conclusions

In an era of enhanced integration of anti-HER2 treatments and while more young patients with early breast cancer are subjected to this class of therapeutic agents in addition to chemotherapy, it is mandatory to evaluate to effect of combined treatment on fertility for optimal patient counseling with regard to reproductive perspectives and treatment options. Our current study sheds light on the potential mechanism by which trastuzumab may lessen chemotherapy-induced vascular toxicity, which leads to ovarian toxicity and potential loss of ovarian reserve. Future prospective clinical and mechanistic studies are warranted to confirm our results.

## Figures and Tables

**Table 1:** Patients characteristics HER2. Abbreviations: Stage—Disease stage at diagnosis.; HER2—Human Epidermal growth factor Receptor 2; chemo—chemotherapy; (−) Negative; DC-H—Docetexal, Carboplatin, Trastuzumab; A—Adriamycin; AC—Adriamycin, Cyclophosphamide; AC-H—Adriamycin, Cyclophosphamide, Trastuzumab; ACT—Adriamycin, Cyclophosphamide, Paclitexal; ACD—Adriamycin, Cyclophosphamide, Docetexal; ACT-H—Adriamycin, Cyclophosphamide, Paclitexal, Trastuzumab; AT—Adriamycin, Paclitexal; DC—Docetexal, Carboplatin; DC-H—Docetexal, Carboplatin, Trastuzumab; T-H—Paclitexal, Trastuzumab.

Patient	Age	Stage	HER2	Chemo Protocol
1	27	1c	3+	DC-H
2	29	1	3+	ACT-H
3	34	3	3+	T-H
4	34	1c	−	ACT
5	36	2	−	AT
6	31	2b	−	AC
7	39	2a	3+	T-H
8	38	1a	3+	AC-H
9	31	1c	−	AC
10	43	1b	3+	DC-H
11	33	1b	−	AT
12	40	2	−	AT
13	37	2	−	AT
14	42	3	3+	T-H
15	37	2a	−	ACD
16	31	2a	−	DC
17	34	3	2	A
18	38	1	2+	DC-H
19	35	2a	1	DC
20	29	1	3+	DC-H
21	35	2a	−	AC
22	26	4	3+	T-H
23	38	2a	3+	T-H
24	27	2b	−	AC
25	37	1b	−	AC
26	38	2a	−	DC
27	29	2	−	AC
28	37	3	−	A
29	28	2	−	AC
30	38	2	3+	ACT-H
31	28	2	3+	T-H
32	36	4	3+	DC-H
33	26	2	−	AC
